# Small Non-Coding RNAs in Human Cancer

**DOI:** 10.3390/genes13112072

**Published:** 2022-11-09

**Authors:** Qunli Xiong, Yaguang Zhang, Junjun Li, Qing Zhu

**Affiliations:** 1 Department of Abdominal Oncology, West China Hospital, Sichuan University, Chengdu 610041, China; 2 State Key Laboratory of Biotherapy and Cancer Center, National Clinical Research Center for Geriatrics, Frontiers Science Center for Disease-Related Molecular Network, West China Hospital, Sichuan University, Chengdu 610041, China; 3 Department of Radiation Oncology, Shanghai General Hospital, Shanghai Jiao Tong University School of Medicine, Shanghai 200080, China

**Keywords:** small non-coding RNA, microRNA, PIWI-interacting RNA, tRNA-derived small RNA, cancer, diagnosis, prognosis, therapy

## Abstract

Small non-coding RNAs are widespread in the biological world and have been extensively explored over the past decades. Their fundamental roles in human health and disease are increasingly appreciated. Furthermore, a growing number of studies have investigated the functions of small non-coding RNAs in cancer initiation and progression. In this review, we provide an overview of the biogenesis of small non-coding RNAs with a focus on microRNAs, PIWI-interacting RNAs, and a new class of tRNA-derived small RNAs. We discuss their biological functions in human cancer and highlight their clinical application as molecular biomarkers or therapeutic targets.

## 1. Introduction

It is estimated that the human genome contains approximately 20,000 protein-coding genes, accounting for only about 2% of the genome sequence [1,2], while the other ~3000 genes, which do not encode proteins and are only transcribed into RNA, are called non-coding RNA (ncRNAs) [3]. For many years, this part of the human genome that does not encode proteins was considered “junk” DNA [4]. It was not until the early 2000s that ncRNAs began to be regarded as potentially crucial regulators of biological processes [5]. These ncRNAs have been reported to exist in almost all fields of life. Small non-coding RNAs (sncRNAs) are a kind of non-coding RNA with less than 200 nucleotides (nt) that exists widely in various prokaryotes and eukaryotic organisms [6,7,8,9]. sncRNAs include classical small-interfering RNAs (siRNAs), microRNAs (miRNAs), PIWI-interacting RNAs (piRNAs), transfer RNAs (tRNAs), small nucleolar RNA (snoRNA), and small nuclear RNA (snRNA), as well as newly discovered non-canonical sncRNAs in the biological world, including tRNA-derived-small RNAs (tsRNAs), vault RNA-derived-small RNAs (vtRNAs), and Y RNA-derived-small RNAs (ytRNAs) [9]. They can regulate cell differentiation, proliferation, migration, angiogenesis, apoptosis, and other crucial biological processes by regulating gene expression during cancer development [10,11], and their dysregulation can trigger homeostatic imbalances that lead to the occurrence and development of diseases such as cancer. Here, we focus on the biological process and roles in tumorigenesis and progression of sncRNAs with length < 50 nt, mainly including classical microRNAs, PIWI-interacting RNAs, and tRNA-derived-small RNAs.

## 2. Biogenesis and Classification

### 2.1. miRNA

**Figure 1:**
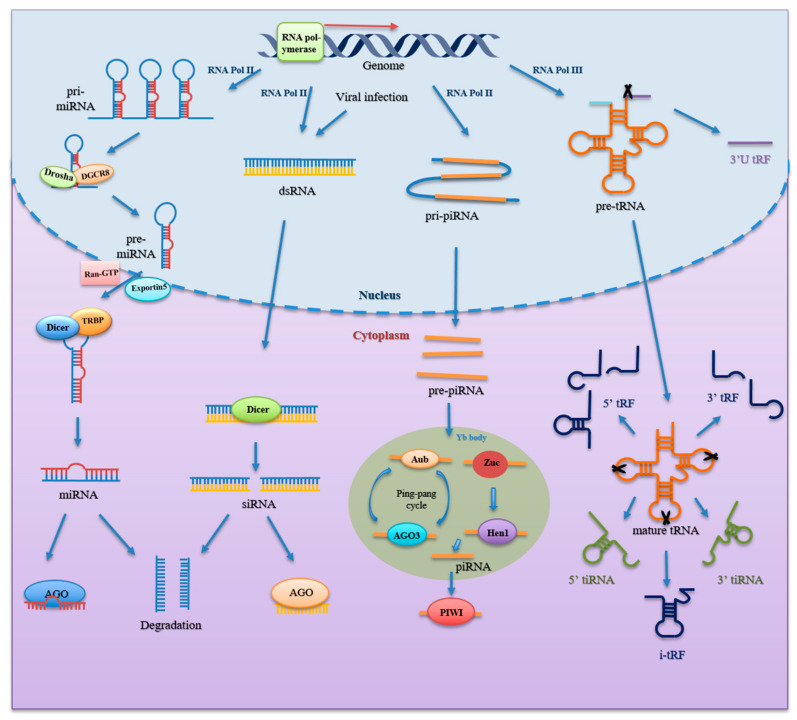
Formation of small non-coding RNAs. miRNA genes are transcribed by RNA polymerase (Pol) II to generate pri-miRNA, which is then processed by DGCR8 and Drosha to form pre-miRNA inside the nucleus. It is further exported to cytoplasm by Exportin5 and Ran-GTP. Dicer and TRBP in cytoplasm further cleave and process pre-miRNA into mature, short double-stranded miRNA. One strand of the mature miRNA duplex binds with Argonaute protein to form RISC, while another strand is degraded. siRNA is derived from long dsRNA produced by transcription of sense and antisense strands by RNA Pol II and viral infection. Then, dsRNA is processed by Dicer into siRNA duplex, of which one strand binds with Argonaute protein to form the siRNA-induced silencing complexes, while another strand is degraded as well. As for piRNA, piRNA genes are transcribed by RNA Pol II to produce pri-piRNA through the primary procession pathway. pri-piRNA is exported into the cytoplasm and cleaved into pre-piRNA. Then, pre-piRNA is processed by Zuc and Hen1 to form mature piRNA. Mature piRNA in complex with PIWI proteins to work. Additionally, Aub and AGO3 coupled with mature piRNA cleave transcript-bearing sites complementary to the piRNA sequence, thus amplifying mature piRNA species through the “ping-pong” cycle. As for tsRNA, after it is transcribed by RNA Pol III, pre-tRNA undergoes processes and modifications to form mature tRNA. Ribonuclease cleavage in various region of pre-tRNA and mature tRNA generates different types of tsRNAs, including 3′U tRF, 5′tRF, 3′tRF, i-tRF, 5′tiRNA, and 3′tiRNA. Abbreviations: miRNA, microRNA; DGCR8, double-stranded RNA-binding protein; pri-miRNA, primary miRNA; pre-miRNA, precursor miRNA; TRBP, TAR RNA binding protein; RISC, RNA- induced silencing complex; siRNA, small interfering RNA; dsRNA, double-stranded RNA; piRNA, PIWI-interacting RNA; pri-piRNA, primary piRNA; pre-piRNA, precursor miRNA; AGO3, Argonaute 3; tsRNA, tRNA-derived small RNA.

microRNA is a class of highly conserved RNAs with 20–25 nt associated with post-transcriptional gene silencing [12]. In 1993, Ambros’ lab discovered the first miRNA from *Caenorhabditis elegans*, LIN-4, an endogenous regulator of genes that control developmental timing [13]. miRNAs are widely present in eukaryotic cells and are one of the most prominent gene families [14]. According to miRBase database, more than 38,000 hairpin precursors and close to 50,000 mature miRNAs from 271 organisms had been identified [15]. miRNA biogenesis is a multistep process tightly regulated by several steps. miRNA genes in the genome are transcribed into primary transcripts (pri-miRNA) by RNA polymerase II [16,17]. Then, primary transcripts are first sheared into hairpin loop-like precursor miRNAs (pre-miRNA) of approximately 70 nt in length by a microprocessor consisting of RNase III (Drosha) and double-stranded RNA-binding protein (DGCR8) [18,19]. Subsequently, pre-miRNAs with characteristic structures can be recognized by the nuclear export protein Exportin-5 (Exp5), which could transport the pre-miRNAs into the cytoplasm through a Ran GTP-dependent mechanism [20]. Then, pre-miRNAs in the cytoplasm are recognized and captured by the Dicer enzyme, resulting in the cleavage of pre-miRNAs by the RNase III domains of the Dicer and TAR RNA binding protein (TRBP) into a double-stranded RNA of about 22 nt in length [21,22,23]. Finally, the double helix structure is unwound, and the miRNA strand and Argonaute (AGO) protein assemble into an RNA-induced silencing complex (RISC), while the remaining strand is degraded [24,25]. A schematic representation of small RNAs biogenesis is presented in Figure 1.

### 2.2. piRNA

piRNA is a single-stranded RNA with a length of 26–31 nt, discovered by scientists in 2006 when extracting and purifying RNA from mouse testis tissue [26,27,28]. piRNA clusters in the genome encode long single-stranded transcripts, named primary piRNAs (pri-piRNAs) [26,29,30]. The process of mature piRNA formation is complex and, as we currently understand it, involves two main pathways: the primary procession pathway and the “ping-pong” cycle [31]. As for the primary processing pathway: piRNA clusters are transcribed into pri-piRNAs by RNA polymerase II and further processed to precursor piRNAs (pre-piRNAs) [30]. The pre-piRNAs are then transported to the cytoplasmic Yb bodies, where they are cleaved by the Zuc riboendonuclease, giving them a 5′-uracil end [32,33]. Subsequently, the 5′ uracil of the pre-piRNA is recognized and bound by the PIWI protein through the PAZ domain to form a piRNA/PIWI complex. After the 3′ end of the piRNA is cleaved by exonuclease, or Zuc, HEN1, or its homolog methylate, the 2′-hydroxyl group at the 3′ end to form the mature piRNA/PIWI complex [34,35,36]. The “ping-pong” cycle is characterized by piRNA/AGO3 or piRNA/Aub complexes. Aub cleaves sense piRNA precursors by coupling to antisense piRNAs, resulting in sense piRNAs loaded onto AGO3. In contrast, antisense piRNA precursors are cleaved by AGO3 with sense strand piRNAs and generate antisense piRNAs that bind to Aub [36,37]. Ultimately, these piRNAs bind to the PIWI protein to work (Figure 1).

### 2.3. tsRNA

tsRNA, ~15–40 nt in length, is produced from precursor tRNAs or mature tRNAs by specific endonucleases activities. tsRNAs were first thought to be random degradation products of tRNAs. Researchers did not find that the expression of certain tsRNAs was stress-induced, as opposed to being the product of random degradation until 2008 [38]. Subsequently, Lee et al. found that tRF-1001, which was the first functional tsRNA discovered, was highly expressed in prostate cancer and affected the growth of prostate cancer cells [39]. Then, its structure and function attracted more attention. According to the cleavage positions and length of the tsRNAs, tsRNAs are classified into three main categories [40]: (1) tiRNA (also called tRNA halves, 31–40 nt), including 5′tiRNA and 3′tiRNA, is produced by specific cleavage within the anticodon loop of mature tRNA by specific enzymes including Angiogenin (ANG), RNase T2, and RNase L under stress conditions [38,39,41,42,43,44,45,46]. 5′tiRNA starts with the 5′end, and 3′tiRNA is derived from the 3′end of mature tRNAs. (2) tRFs (14–30 nt) include 5′tRF, 3′tRF and inter tRF (i-tRF) [47,48]. 5′tRF originates from cleavage in in D-loop, while 3′tRF is mainly cleaved in T-loop. The i-tRFs are derived from the internal region of mature tRNAs, around the anticodon. (3) 3′U tRFs (20–40 nt) are derived from cleavage in 3′ end of precursor tRNAs (Figure 1).

### 2.4. siRNA

Small interfering RNAs are double-stranded RNA molecules 20 to 25 nt in length [12]. In 1999, scientists discovered that transgenic and virus-induced plant silencing is accompanied by the emergence of RNAs of ~20–25 nt that match the sequence of the targeted mRNA [49]. The siRNA was then recorded. It is derived from splicing of long, complementary double-stranded RNA (dsRNA) formed by Dicer [12]. One of the strands binds to the AGO protein to form a functional RISC, which can recognize and bind mRNAs with complementary sequences, while another chain is degraded [12,50]. In many species, target-bound siRNAs can enhance and maintain silencing responses via amplifying by RNA-dependent RNA polymerase (RdRP), while mammalian genomes lack RdRP-encoding potential. Although some genomically derived siRNAs have been identified in mammals [51], most of the characterized siRNAs are either virally encoded or experimentally induced. Therefore, in mammalian cells, including those of humans, the classical RNA interference pathway via siRNA is considered to be mainly used for defense against external threats. A schematic representation of small RNAs biogenesis is presented in Figure 1.

### 2.5. Other sncRNAs

Aside from the above-mentioned small non-coding RNAs, recently discovered non-canonical sncRNAs derived from structural RNAs are emerging, such as rsRNA (from ribosomal RNA) [52], ysRNA (from Y RNA) [52,53], snsRNA (from small nuclear RNA) [54,55], snosRNA (from small nucleolar RNA) [56], and vtsRNA (from vault tRNA) [57]. Compared with classical small RNAs including siRNA, miRNA, and piRNA, non-canonical small RNAs have distinct features in terms of origin, biogenesis, abundance, and function, which may update our understanding of sncRNAs [9,40]. Nevertheless, the research on the generation and function of these sncRNAs is still in its infancy and needs to be explored.

## 3. Functions of sncRNAs

As sncRNAs is increasingly studied, its function becomes clearer than before. They regulate various biological processes mainly through transcriptional, post-transcriptional, translational, post-translational, and reverse transcriptional regulation.

### 3.1. Transcriptional Regulation

After the small RNA matures in the cytoplasm, some small RNAs re-enter the nucleus to regulate the transcription level. The piRNA/PIWI complex can recruit the cofactor Panoramix/Silesio (panx) or the epigenetic factor HP1a and the necessary histone modifying enzymes to the modification site to maintain the inhibitory state of chromatin [58,59]. Another small noncoding RNA member, tsRNA, can regulate genome expression in a PIWI-dependent manner through a mechanism similar to that of piRNA. For example, Zhang et al. found that a piRNA-like tsRNA, 5′tRH-Glu-CTC, could bind to PIWIL4 to form a complex in human monocytes or dendritic cells, methylating H3K9 and repressing *CD1A* transcription by recruiting H3K9 methyltransferase and heterochromatin protein 1b to the *CD1A* promoter region [60]. In addition to transcriptional gene silencing (TGS), small RNAs are involved in transcriptional gene activation (TGA). The nuclear-enriched miR-195-5p increased Foxo3 expression by targeting the *Foxo3* promoter, which may be engaged in AGO2 recruitment and histone demethylation and acetylation in cells [61]. Apart from the promoter, small RNAs can regulate transcription at enhancers as well. For example, elevation of miR-26a-1 leads to enhanced transcriptional of *ITGA9* and *VILL* genes by targeting their enhancers [62]. Overexpression of miR-24-1 resulted in the increase of RNA polymerase II, p300/CBP, and enhancer RNA [63]. In summary, these studies suggest a regulatory function of small RNAs for transcriptional regulation at the enhancer and promoter level.

### 3.2. Post-Transcriptional Regulation

In mammals, RNA-induced silencing complex (RISC) gene silencing is a well-known mechanism of cytoplasmic post-transcriptional gene regulation. Typically, small RNAs target specific sites in mRNAs to engage in their post-transcriptional regulation in the form of mRNA degradation or translational inhibition. This process relies on the recognition of target mRNAs by small RNAs. In a typical RNAi pathway, the siRNA guide strand directs RISC to a fully complementary RNA target. Guided by the siRNA, RISC cleaves mRNA in the mRNA-siRNA duplex, and the resulting mRNA is degraded [6]. miRNA binding sites in mammalian mRNAs are located in the 3′UTR region in most cases. Most animal miRNAs bind with mismatches and bulges, although miRNA nucleotides 2–8 form seeds critical for the recognition of target mRNAs [12], leading to translation inhibition. When the miRNA acts in compete complementarity with the target gene, its mode of action and function is very similar to that of siRNA, resulting in the instability and degradation of mRNA. Cytosolic polyadenylate-binding protein (PABPC) is recruited through the functions of AGO protein and glycine-tryptophan protein (GW182), promoting mRNA deadenylation [64,65]. The mRNA is then rapidly degraded after uncapping by the DCP1-DCP2 complex [66]. Additional small non-coding RNAs, piRNAs, and tsRNAs, can also repress target gene function through regulatory post-transcriptional networks similar to miRNAs. In contrast, piRNA interactions require strict base pairing within 2–11 nt and less stringent base pairing within 12–21 nt of the 5′ end of the piRNA [67]. For example, piR-55490 targets the 3′UTR region of mTOR mRNA, resulting in mRNA degradation and the inhibition of cell growth and proliferation in lung cancer [68]. CU1276, a miRNA derived from tRNA-Gly-GCC, could bind to AGO protein and target the 3′UTR of mRNA in normal germinal center B cells to inhibit gene expression [69]. Furthermore, miR-3676 targets three consecutive 28-bp repeats within the 3′UTR of TCL1 mRNA, thereby inhibiting the translation of TCL1 [70]. In addition, small RNAs can also undergo post-transcriptional regulation through other pathways. For example, piR-30840 is crucial to the development of Th2 lymphocytes by suppressing the maturation of interleukin-4 mRNA [71]. tsRNAs can act as protein decoys, sequestering RNA-binding proteins from targeted RNAs, thereby influencing RNA stability [72,73]. The above mechanisms provide new insights into how small non-coding RNAs regulate gene expression in the post-transcriptional level.

### 3.3. Translation and Post-Translation Regulation

tsRNA can interact with ribosomal subunits in a similar manner to its precursor, mature tRNA, thereby affecting translation [74]. Gebetsberger et al. reported that Val-tRF is able to compete with mRNA rather than tRNA for ribosomal 30S subunits, affecting translation initiation to inhibit translation [75]. Ivanov et al. found that tiRNAs directly or indirectly bind to eIF4G, eIF4A, or eIF4G/A complexes, thereby inhibiting translation. In addition, tiRNAs bind to YB-1, which together prevent eIF4G/A from initiating translation [76]. Shi et al. illustrated that 5′tsRNA-Ala and –Cys with a terminal oligo g motif could form into G-quadruplex-like structure, which can competitively bind to translation-initiation factor eIF4E/G/A and then suppress mRNA translation [77]. In addition, studies have shown that small RNAs can also facilitate translational processes. For example, tRNA-Leu-CAG derived small RNA binds to the coding region CDS and the non-coding 3′UTR region of ribosomal protein S28 (RPS28), altering the secondary structure to increase its translation, while inhibiting this tsRNA reduces RPS28 protein levels, suggesting that 3′ tsRNA-Leu-CAG may maintain ribosome biosynthesis through a conserved gene regulatory mechanism [78,79]. Furthermore, Fricker et al. showed that the tRNA-Thr 3′half produced by *Trypanosoma brucei* during nutrient deprivation could bind to ribosomes and multimers, stimulated by promoting mRNA loading during stress recovery after starvation conditions have ceased. Translation, blocking or depletion of endogenous tRNA-Thr halves alleviated this stimulatory effect in vivo and in vitro [80].

Small RNAs can modulate the structure and function of interacting proteins by binding to translation-independent proteins. For example, 21 nt i-tRF-Gly interacts with hnRNPL and then prevents phosphorylation of hnRNPL by AKT2, thereby reducing the AKT2-hnRNAPL-DDX17 axis and attenuating the malignant phenotype in pancreatic cancer cells [81]. Zhao et al. found that piRNAs regulate the ubiquitination of the PIWI protein Miwi by enhancing the interaction of Miwi with the substrate-binding subunit of APC/C during late mouse spermatogenesis [82]. Yin et al. found that piR-823 could upregulate the transcriptional activity of the heat shock factor 1(HSF1) by interacting with HSF1 and promoting its phosphorylation at Ser326 [83]. Mai et al. reported that piR-54265/PIWIL2 could recruit STAT3 and phosphorylated-SRC to form a complex through the PIWIL2 PAZ domain, facilitating phosphorylated-SRC-mediated STAT3 phosphorylation and activation of related signaling pathways [84]. The direct binding of piRNAs/PIWI complexes to specific proteins via piRNAs or the PIWI protein PAZ domain can alter their subcellular localization [85].

### 3.4. Reverse Transcription Regulation

Viruses rely on the host cell’s translation machinery to efficiently synthesize their own proteins. Many RNA viruses and retrotransposons can self-replicate by using the 3′ end of mature tRNA as a primer for their reverse transcription (RT). Emerging evidence suggests that specific host tRNA fragments can be used as primers for reverse transcription and packaged into retroviruses [86]. For example, Ruggero et al. demonstrated that tRF-3019 has good sequence complementarity with the primer binding site of human T-cell leukemia virus type 1 (HTLV-1), and the results of in vitro reverse transcriptase tests confirmed that tRF-3019 can trigger HTLV-1 reverse transcription [87]. This suggests that tRF-3019, as a reverse transcription primer, plays an important role in initiating reverse transcription of HTLV-1 and may become a new target for controlling HTLV-1 infection. Additionally, Schorn et al. showed that exogenous 18 nt 3′ tsRNA could target the tRNA primer binding sites necessary for endogenous retroviral reverse transcription, IAP and MusD/ETn, resulting in strong reverse transcription inhibition [88]. These studies show that the viral world adapts to specific tsRNAs that can block or mimic the function of tRNAs and thus can be potential therapeutic targets.

## 4. Functions of Small Non-Coding RNAs in Cancer

Cancer is thought to be characterized by a series of functional capabilities that human cells acquire during the transition from a normal state to a tumor-growing state that is critical to the ability to form malignancies. In 2002, Calin et al. found that miR-15 and miR-16 were absent and downregulated frequently in chronic lymphocytic leukemia, presenting evidence for the involvement of miRNA genes in human cancer progression [89]. The importance of tsRNA in the progression of human cancer was first illustrated in prostate cancer. In 2009, Lee et al. created and sequenced a small RNAs library in prostate cancer cell lines, finding that Trf-1001, a tsRNA derived from pre-tRNA-Ser, could accelerate the growth of prostate cancer [39]. In past studies, almost all known cancer occurrence or development has been associated with small RNAs (Table 1). These small RNAs are closely associated with cancer hallmarks reported by Hanahan [90,91,92], including maintaining proliferative signaling, resisting cell death, inducing or accessing angiogenesis, tumor-promoting inflammation, activating invasion and metastasis, etc. Here, we mainly focus on four recently proposed cancer hallmarks, which encourage us to study small RNAs in terms of other features of cancer.

### 4.1. Unlocking Phenotypic Plasticity

Usually, cellular differentiation leads to a distinct barrier to continued proliferation necessary for tumorigenesis. Growing evidence suggests that unlocking the restricted ability of phenotypic plasticity to avoid a terminally differentiated state is significant for tumor development [91,114]. Overexpression of miR-4423 induced a differentiation-like pattern of gene expression in airway epithelial cells and reversed the expression of some genes that were altered in lung cancer, i.e., ablation of miR-4423 function contributed to the development of lung cancer [115]. Overexpression of miR-17-92 cluster in normal thyroid follicular cells could impair thyroid differentiation and promote cancer-related effects by inhibiting TGF-β signaling [116]. Upregulation of miR-34a expression was observed at the same time as the induction of osteosarcoma cells into a stem cell-like phenotype. Further study found that plasminogen activator inhibitor-1 (PAI-1) was the downstream of miR-34a, and suppressing PAI-1 could inhibit osteosarcoma dedifferentiation into cancer stem-like cells by blocking Sox2 expression [117]. miR-215 could promote the self-renewal of colorectal cancer stem cells via the targeted gene *BMI1* [118]. miRNA-302a/d could downregulate the self-renewal ability of liver cancer stem cells and proliferation of liver cancer cells by inhibiting E2F7/AKT/β-catenin/CCND1 signaling pathway [119]. These studies suggest that small RNAs play an important role in unlocking tumor phenotypic plasticity.

### 4.2. Non-Mutational Epigenetic Reprogramming

In 2012, Huang proposed the conception of mutation-less cancer evolution and purely epigenetic programming of hallmark cancer phenotypes [91,120] and is increasingly concerned subsequently [121,122,123]. A growing body of research supports small RNAs as novel epigenetic regulators, contributing to the acquisition of multiple hallmark abilities during cancer initiation and progression. piRNAs have been reported to be epigenetic effectors in human tumors [124]. piRNA-30473 is involved in tumorigenesis in diffuse large B-cell lymphoma by upregulating m6A mRNA methylase WTAP [110]. In addition, sperm tsRNA can be an epigenetic factor that mediates the intergenerational inheritance of diet-induced metabolic disorders [125]. Zygote injection of individual tsRNAs or a combination of tsRNAs in early embryo stages could induce alterations gene expression, indicating that sperm tsRNAs transmit paternally acquired traits via remodeling early embryonic development [125,126].

### 4.3. Polymorphic Microbiomes

Polymorphic variation in microbial communities between individuals has noticeable influences on malignant behaviors [127,128]. Numerous researchers have demonstrated an association between gut microbiomes and cancer [129,130], especially colorectal cancer. Several studies have shown that various subtypes of colorectal cancer (CRC) are linked to distinct microbiome “fingerprints” [131]. Other studies have reported a specific accumulation of seemingly cancer-related microorganisms in CRC. MicroRNAs (miRNAs) influence key cellular processes and are closely related to CRC progression. A growing body of research has found that miRNAs can mediate bidirectional interactions between microbiome and host. Of note, the gut microbiome can regulate miRNA expression to alter the host transcriptome, affecting the progression of CRC [132]. One microorganism closely related to colorectal cancer is *Fusobacterium nucleatum*. This bacterium upregulates miR-21 in cancer by activating the tlr4-myd88 signaling pathway, decreasing protein levels of tumor suppressor genes *RASA1* and *PDCD4*, thereby promoting CRC growth [133]. *Helicobacterpylori* ((*H. pylori*) infection also can affect host miRNAs expression. Chang et al. reported that miR99b-3p, miR-564, and miR-638 significantly upregulated, while miR-204-5p, miR-338-5p, miR-375, and miR-548c-3p downregulated in *H. pylori*-positive gastric cancer patients, compared with *H. pylori*-negative patients [134]. Furthermore, the relationship between vaginal microbiota, miRNAs and ovarian cancer was confirmed as well. For example, vaginal isolated *Lactococcus lactis* can decrease TLR-4, miR-21, and miR-200b expression [135], among which the miRNA-21 and miR-200 family were found to be connected with cancer metastasis, and overall survival rate in ovarian cancer [136,137].

### 4.4. Senescent Cells

Cellular senescence is a typical irreversible proliferation arrest that may evolve into a protective mechanism to maintain tissue homeostasis [91]. Senescence has long been protective in limiting malignant progression. That is, cancer cells are induced to undergo senescence to inhibit tumor progression [138]. For example, miR-130b~301b overexpression effectively induces prostate cancer cell growth arrest through activation of cellular senescence [139]. miR-30 could decrease cell senescence and induce cancer progression by inhibiting CHD7 and TNRC6A [140]. Epstein–Barr virus-encoded miR-BART3-3p has been reported to accelerate tumorigenesis by inhibiting the senescence process in gastric cancer [141]. miR-7 could repress the gemcitabine-induced senescence by targeting PARP1/NF-κB pathway in pancreatic cancer [142]. However, in some cases, senescent cells promote cancer progression differently [143,144]. Its primary mechanism is senescence-associated secretory phenotype (SASP), involving the release of a large number of biologically active proteins containing prominent pro-inflammatory cytokines and chemokines [145], which can transmit signals in a paracrine manner to nearby living cancer cells and other cells in the TME, thereby promoting tumor progression [146,147].For example, upregulated miR-335 in senescent normal fibroblasts and cancer-associated fibroblasts regulates SASP factor secretion and induces cancer cell motility in co-cultures by inhibiting PTEN expression [148]. However, the mechanism of this small RNA-related senescence-promoting tumor still needs more research to support it.

## 5. Clinical Significances of Small Non-Coding RNAs in Cancer

Increasing evidence indicates that small non-coding RNAs are differentially expressed in tumor tissue or the circulating blood of tumor patients and play a crucial regulatory role in tumor initiation and progression. It can reflect the presence of the tumor, pathological grading, clinical staging, and clinical outcome of the patient. This suggests that small non-coding RNAs have great potential to serve as cancer diagnosis, prognosis biomarkers, and therapeutic targets.

### 5.1. Diagnostic and Prognostic Biomarker

The miR-1290 is significantly upregulated in the blood of colorectal adenoma and colorectal adenocarcinoma patients. Blood miR-1290 levels can distinguish adenomas, CRC patients and normal subjects, suggesting that serum miR-1290 is a potential diagnostic biomarker for colorectal cancer. At the same time, the high expression of miR-1290 is closely linked to cancer aggressiveness and poor outcome. It is an independent factor for prognosis of patients with colorectal cancer [149]. Serum tRF-Pro-AGG-004 and tRF-Leu-CAG-002 is significantly elevated in pancreatic cancer patients compared with healthy people, suggesting that they are promising diagnostic biomarkers. Additionally, in situ hybridization (ISH) scores in tumor tissues suggest that tRF-Pro-AGG-004 and tRF-Leu-CAG-002 can predict postoperative survival time of pancreatic cancer patients [113]. Furthermore, small RNAs can be novel diagnostic and prognostic biomarkers in other cancer types, including ovarian cancer [150], breast cancer [151], prostate cancer [152], etc. Notably, exosome-derived small non-coding RNAs can also serve as useful diagnostic and prognostic biomarkers [153,154,155,156]. For example, tRNA-GlyGCC-5 and another small non-coding RNA, sRESE, are enriched in saliva-derived exosomes from esophageal squamous cell carcinoma patients compared to healthy controls, and the bi-signature containing these two small non-coding RNAs can distinguish esophageal carcinoma patients from the controls with a high sensitivity of 90.50% and specificity of 94.20%. Furthermore, patients with a high bi-signature have both poorer overall survival and progression-free survival [155]. In endometrial carcinoma, a plasma-derived exosomal microRNA, miR-15a-5p, can distinguish cancer patients from controls with an Area Under Curve (AUC) value of 0.813. Of note, the combination of miR-15a-5p with serum CEA and CA125 could achieve a higher AUC value of 0.899 [156]. These studies provide unique insights and more dynamic perspectives of cancer diagnosis and prognosis. Research on the use of small non-coding RNAs as molecular biomarkers for cancer diagnosis or prognosis is presented in Table 2.

### 5.2. Therapy

Some studies have suggested that small non-coding RNAs can serve as functional molecules linked to cancer initiation and progression, as well as functioning as promising therapeutic targets or agents. For example, miR-1293 can inhibit tumor cell growth in vitro by inhibiting DNA repair genes and BRD4, a member of the Bromodomain and Extra-terminal Domain (BET) protein family, is recognized as a promising target for cancer therapy [198]. miRNAs can also be combined with existing treatments, such as chemotherapy [199,200], radiotherapy [201,202], and immunotherapy [203,204] to treat tumors synergistically. For example, Temozolomide (TMZ)-resistant glioblastoma cells confer TMZ chemoresistance to receptor TMZ-sensitive cells in an exosomal miR-151a loss-dependent manner, so miR-151a mimics transfected into glioblastoma cell lines can sensitize TMZ-resistant GBM cells via inhibiting XRCC4-mediated DNA repair [199]. miR-205 is highly downregulated in gemcitabine (GEM)-resistant pancreatic cancer cells and can resensitize GEM-resistant pancreatic cancer cells to GEM [200]. In addition, miRNAs can also be used for “replacement therapy”, which aims to restore the expression of repressor miRNAs [205]. For example, the treatment of miR-34a and let-7b in neuroblastomas significantly reduces cell division, proliferation, neo-angiogenesis, and induces apoptosis in orthotopic xenografts [206]. Furthermore, other small RNAs also show good therapeutic prospects. For example, inhibition of 3′tsRNA-Leu-CAG promotes apoptosis in rapidly dividing cells in vitro and in vivo [78]. piRNA-36712 has synergistic anti-cancer effects with paclitaxel and doxorubicin in breast cancer [103]. However, the application of these small RNAs to clinical practice still requires a lot of effort.

## 6. Conclusions and Prospects

With the development of high-throughput sequencing technology, more and more sncRNAs have been discovered. With the deepening of sncRNA function research, its essential role in cancer has gradually emerged. Aberrant expression of various sncRNAs is an important regulator of activating or affecting signaling pathways during cancer development, making sncRNAs’ expression a diagnostic and prognostic marker and a potential therapeutic target. However, there is still much to learn surrounding the exact roles of various sncRNAs in cancer. The mechanism of action of sncRNAs in tumors needs more relevant studies to fully understand their mechanism of action and promote the development of this field of cancer biology.

## Figures and Tables

**Table 1:** Functions of small non-coding RNAs related to human cancer.

Cancer Type	Small RNA (Examples)	Expression	Function	Mechanism	References
Gastric cancer	miR-371-3	Up	Promotes cell proliferation, migration and invasion	Suppresses TOB1	[93]
	piR-823	Down	Inhibits cell growth	Unknown	[94]
	tRF-5026a	Down	Inhibits cell proliferation, migration, and cell cycle	Regulates the PTEN/PI3K/AKT signaling pathway	[95]
Liver cancer	miR-802	Up	Promotes cell proliferation, cell cycle and migration	Suppresses RUNX3	[96]
	piR-Hep1	Up	Promotes cell viability, motility, and invasiveness	Reduces phosphorylated AKT	[97]
	Gly-tRF	Up	Promotes liver cancer stem cell-like cell properties and cell migration	Suppresses NDFIP2 and increases phosphorylated AKT	[98]
Colorectal cancer	miR-4319	Down	Inhibits cell proliferation, migration and invasion	Suppresses ABTB1	[99]
	piR-823	Down	Inhibits cell apoptosis	Interacts with PINK1	[100]
	5′tiRNA-His-GTG	Up	Promotes cell proliferation	Suppresses LATS2	[101]
Breast cancer	miR-21	Up	Promotes cell proliferation and metastasis	Suppresses LZTFL1	[102]
	piRNA-36712	Down	Inhibits cell proliferation and cell cycle	Interacts with SEPW1P RNA	[103]
	5′-tiRNA-Val	Down	Inhibits cell proliferation and migration	Inhibits FZD3/Wnt/β-Catenin signaling pathway	[104]
Lung cancer	miR-1254	Up	Promotes cell proliferation	Suppresses SFRP1	[105]
	tsRNA-5001a	Up	Promotes cell proliferation	May target GADD45G	[106]
Multiple myeloma	miR-145-3p	Down	Inhibits cell proliferation and promotes apoptosis	Suppresses HDAC4	[107]
	piRNA-823	Up	Promotes cell proliferation, apoptosis and cell cycle	Regulates de novo DNA methylation and angiogenesis	[108]
Lympho-ma	miR-339-5p	Up	Promotes cell proliferation, cell cycle and reduces apoptosis	Suppresses BCL2L11 and BAX	[109]
	piRNA-30473	Up	Promotes cell proliferation and cell cycle	Regulates m6A RNA methylation	[110]
Pancreatic cancer	miR-4516	Down	Inhibits cell proliferation, migration and invasion, while promotes cell apoptosis	Suppresses OTX1	[111]
	piR-017061	Down	inhibits cell growth	Suppresses EFNA5	[112]
	tRF-Pro-AGG-004 and tRF-Leu-CAG-002	Up	Promotes cell growth and invasion	unknown	[113]

**Table 2:** Small non-coding RNAs as molecular biomarkers for cancer diagnosis or prognosis.

Cancer Type	Diagnosis	Prognosis
Lung cancer	miR-20a, miR-10b, miR-150, miR-223, miR-205 [157]; 5a_tRF-Ile-AAT/GAT, 5a_tRF-Asp-GTC, 3P_tRNA-Arg-TCG-1-, 3P_tRNA-Arg-TCT-4-1, 5P_tRNA-Gly-TCC-1-1, 5P_tRNA-Asn-GTT-2-3 [158]; piR-651 [159]	miR-21, miR-155, miR-200c and miR-125b, miR-21, miR-155, miR-200c and miR-125b [160]; AS-tDR-007333 [161], 5a_tRF-Ile-AAT/GAT [158]; piR-651 [159]
Prostate cancer	miR-26b-5p, miR-98-5p [162], miR-5100, miR-1290 [163]	miR-182 [164]; miR-1290, miR-375 [165]
Gastric cancer	miR-140, miR-183, miR-30e, miR-103a, miR-126, miR-93, miR-142, miR-21, miR-29c, miR-424 and miR-181a, miR-340 [166]; miR-21, miR-222, miR-99a-5p; miR-17, miR-25, miR-214 [167]; miR-4257, miR-6785-5p, miR-187-5p, and miR-5739 [168]; tRF-23-Q99P9P9NDD [169]	miR-501-5p, miR-208a, miR-718, miR-15b-3p, miR-519a, miR-153, miR-187, miR-345, miR-28-5p, miR-124-3p, miR-383-5p [167]; tRF-23-Q99P9P9NDD [169]
Colorectal cancer	miR-21, miR-24-2, miR-139-3p, miR-135a-5p, miR-21, miR-29, miR-92, miR-125, miR-223, miR-6803-5p, miR-21, miR-4478 [170]; tRF-phe-GAA-031, tRF-VAL-TCA-002 [171]	miR-21, miR-6826, miR-122, miR-139-5p, miR-203, miR-6803-5p, miR-17-5p [170]; miR-5091, miR-10b-3p, miR-9-5p, miR-187-3p, miR-32-5p, miR-652-3p, miR-342-3p, miR-501-3p, miR-328-3p [172]; tRF-phe-GAA-031, tRF-VAL-TCA-002 [171]; 5′-tiRNA-Pro-TGG [173]
Liver cancer	miR-10b-5p, miR-221-3p, miR-223-3p, miR-21-5p [174]; tRNA-ValTAC-3, tRNA-GlyTCC-5, tRNA-ValAAC-5; tRNA-GluCTC-5 [153]; tRF-Gln-TTG-006 [175]	miR92a-3p [176], miR-125b [177], miR-144, miR-451a [178]; miR-638 [179]
Pancreatic cancer	miR-21, miR-155, miR-196a, miR-196b, miR-16, miR-18a, miR-20a [180], let-7b-5p, miR-192- 5p, miR-19a-3p, miR-19b-3p, miR-223-3p, and miR-25-3p [181]; tRF-Pro-AGG-004, tRF-Leu-CAG-002 [113]	miR-10b, miR-21, miR-34a, miR-155, miR-200 family, miR-216, let-7 family [182]; tRF-Pro-AGG-004, tRF-Leu-CAG-002 [113]
Ovarian cancer	miR-4732-5p [183]; miRNA-205 [184]; miR-21, miR-200 family, miR-205, miR-10a, miR-346 [185]	miRNA-150-5p [186]; miR-508-3p [187]; miR-23a-3p [188]; miR-203 [189]; i-tRF-Gly-GCC [190]
Glioma	miR-155-5p [191]; miR-454-3p [192]; miRNA-21[193]	miR-10b, miR-222 [194]; miR-4476 [195]; miR-454-3p [192]
Breast cancer	miR-21, miR-96, miR-183, miR-182, miR-141, miR-200a, miR-429, miR-139, miR-145 [196]; RF-Arg-CCT-017, tRF-Gly-CCC-001, tiRNA-Phe-GAA-003 [197]	miR-17, miR-18a, miR-19a, miR-19b, miR-20a, miR-27b-3p, miR-92, miR-497, miR-532-5p [196]; piRNA-36712 [103]; tRF-Arg-CCT-017, tiRNA-Phe-GAA-003 [197]

## Data Availability

Not applicable.
